# Fundamental Neurochemistry Review: Lipids across microglial states

**DOI:** 10.1111/jnc.16259

**Published:** 2024-12-18

**Authors:** Marianela E. Traetta, Haley A. Vecchiarelli, Marie‐Ève Tremblay

**Affiliations:** ^1^ Division of Medical Sciences University of Victoria Victoria British Columbia Canada; ^2^ Centre for Advanced Materials and Related Technology (CAMTEC) University of Victoria Victoria British Columbia Canada; ^3^ Institute for Aging and Lifelong Health (IALH) University of Victoria Victoria British Columbia Canada; ^4^ Département de médecine moléculaire Université Laval Québec City Quebec Canada; ^5^ Axe neurosciences, Centre de recherche du CHU de Québec Université Laval Québec City Quebec Canada; ^6^ Neurology and Neurosurgery Department McGill University Montréal Quebec Canada; ^7^ Department of Biochemistry and Molecular Biology University of British Columbia Vancouver British Columbia Canada

**Keywords:** immunometabolism, lipid, microglia, microglial states

## Abstract

The capacity of immune cells to alter their function based on their metabolism is the basis of the emerging field of immunometabolism. Microglia are the resident innate immune cells of the central nervous system, and it is a current focus of the field to investigate how alterations in their metabolism impact these cells. Microglia have the ability to utilize lipids, such as fatty acids, as energy sources, but also alterations in lipids can impact microglial form and function. Recent studies highlighting different microglial states and transcriptional signatures have highlighted modifications in lipid processing as defining these states. This review highlights these recent studies and uses these altered pathways to discuss the current understanding of lipid biology in microglia. The studies highlighted here review how lipids may alter microglial phagocytic functioning or alter their pro‐ and anti‐inflammatory balance. These studies provide a foundation by which lipid supplementation or diet alterations could influence microglial states and function. Furthermore, targets modulating microglial lipid metabolism may provide new treatment avenues.

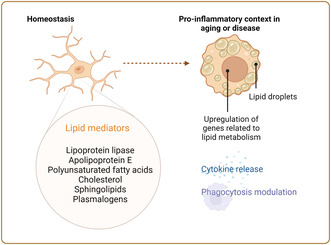

## Graphical abstract

In this review we described the current understanding of lipid biology in microglia, highlighting how lipids may alter microglial phagocytic functioning or inflammatory balance. Recent studies describe different microglial states, with transcriptional signatures that have highlighted modifications in lipid processing, including foamy and lipid droplet‐accumulating microglia. We addressed commonly altered lipid pathways, using those that are altered in these states as a guide to provide a detailed description of how they impact microglial function. We summarized the actions of lipoprotein lipase, apolipoprotein E, polyunsaturated fatty acids, cholesterol, sphingolipids and plasmalogens on microglial structure, metabolism, and function.

**Abbreviations**
ABCA1: ATP binding cassette subfamily A member 1
ABCG1: ATP binding cassette subfamily G member 1
Acetyl‐CoA: acetyl coenzyme A
ACOX: acyl‐CoA oxidase
ACSL1: adipose acyl‐CoA synthetase‐1
AD: Alzheimer's disease
APOC1: apolipoprotein C1
APOE: apolipoprotein E
ATP: adenosine triphosphate
Aβ: amyloid beta
BDNF: brain‐derived neurotrophic factor
CD: cluster of differentiation
Ch25h: cholesterol 25‐hydroxylase
CNS: central nervous system
CPT1a: carnitine palmitoyltransferase 1a
CTSB: cathepsin B
CTSD: cathepsin D
CX3CR1: CX3C motif chemokine receptor 1/fractalkine receptor
DAM: disease‐associated microglia
DHA: docosahexaenoic
EPA: eicosapentaenoic
FABP5: fatty acid binding protein 5
FADH: flavin adenine dinucleotide
FFA: free fatty acids
GFAP: glial fibrillary acidic protein
GLUT: glucose transporter
GPNMB: transmembrane glycoprotein NMB
GRN: progranulin
HDL: discoidal high‐density lipid
HETE: hydroxyeicosatetraenoic acid
HMGB1: high mobility group box 1
IBA1: ionized calcium‐binding adapter molecule 1
IFNγ: interferon γ
IL1β: interleukin 1β
IL4: interleukin 4
iPSC: induced pluripotent stem cell
JAK–STAT: Janus‐kinase and signal transducer and activator of transcription proteins
LAMP1/2: lysosomal‐associated membrane protein
LDAM: lipid droplet‐accumulating microglia
LDLR: low‐density lipoprotein receptor
LIPA: lipase A, lysosomal acid type
LOX: lipoxygenase
LPL: lipoprotein lipase
LPS: lipopolysaccharide
LRP1: LDLR‐related receptor 1
LXR: liver X receptor
MALDI: matrix‐assisted laser desorption/ionization
MFP2: multifunctional protein‐2
MGnD: microglial neurodegenerative phenotype
MMP: matrix metalloproteinase
mTOR: mammalian target of rapamycin
NADH: nicotinamide adenine dinucleotide
NCEH1: neutral cholesterol ester hydrolase 1
NeuN: neuronal nuclear protein
NF‐kB: nuclear factor kappa B
NPC: Niemann‐Pick type C
P: postnatal day
PAM: proliferative‐region‐associated microglia
PlsCho: choline plasmalogen
PlsEtn: ethanolamine plasmalogen
PUFA: polyunsaturated fats
RNA: ribonucleic acid
ROS: reactive oxygen species
scRNA‐seq: single‐cell RNA‐sequencing
SNAT1: sodium‐coupled neutral amino acid transporter 1
snRNA‐seq: single nucleus RNA‐sequencing
SOAT1: sterol O‐Acyltransferase 1
TLR: Toll‐like receptor
TMEM119: transmembrane protein 119
TNF: tumor necrosis factor
TREM2: triggering receptor expressed on myeloid cells 2
TSPO: 18‐kDa translocator protein
TYROBP: tyrosine kinase‐binding protein

## 1 MICROGLIA AND THEIR METABOLISM

Microglia are the resident innate immune cells in the central nervous system (CNS). They are incredibly important for CNS health throughout the lifespan and are implicated in almost all neurological diseases. These cells support and regulate all other cell types in the CNS, including astrocytes, oligodendrocytes, and neurons, as well as serve to modulate the physical components of the CNS, such as blood vessels and the blood–brain barrier (Paolicelli et al., 2022; Sierra et al., 2019; Šimončičová et al., 2022; Tay et al., 2019; Vecchiarelli et al., 2021; Vecchiarelli & Tremblay, 2023a, 2023b). In order to accomplish these regulatory functions, which can notably involve the release of inflammatory mediators and trophic factors and/or phagocytosis, microglia must tightly regulate their metabolic profile (Bernier et al., 2020). An emergent field of study, immunometabolism, aims to determine how metabolism shapes the function of immune cells. It is currently a focus of the microglial field to illuminate these cells' immunometabolism, particularly since their metabolic reprogramming has been linked to neurodegenerative diseases (reviewed in Miao et al. (2023)), but is presumably also important to many health and disease contexts.

**FIGURE 1:**
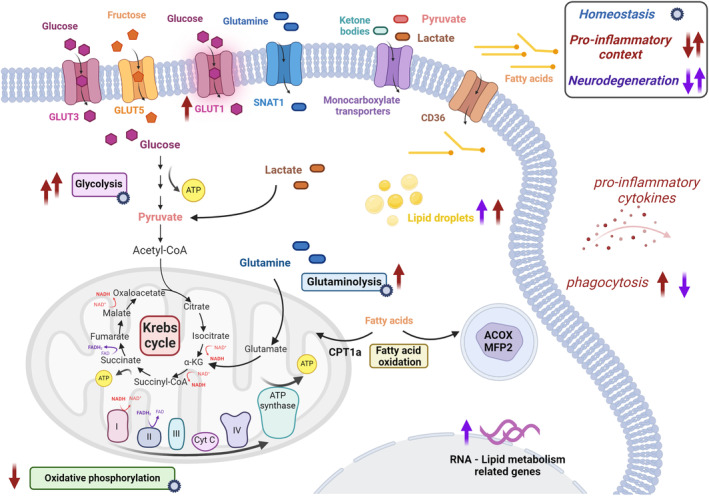
Microglial metabolism. Under homeostatic conditions, microglia can use numerous fuel sources. They express glucose transporters and obtain ATP through glycolysis and oxidative phosphorylation. Microglia can also internalize glutamine and generate ATP through glutaminolysis. Fatty acid oxidation does not seem to be a main source of energy under homeostatic conditions. In a pro‐inflammatory context, glycolysis is increased, while the expression of other glucose transporters enhancing glucose incorporation and oxidative phosphorylation is reduced, with the Krebs cycle being compromised. These metabolic changes are accompanied by an increase in the secretion of pro‐inflammatory cytokines and phagocytic activity. In the context of neurodegeneration, there are some microglial states in which the transcriptomic profile is characterized by an increase in genes related to lipid metabolism, along with an increase in lipid droplets and a decrease in their phagocytic activity. ACOX, acyl‐CoA oxidase, CD36, cluster of differentiation 36; CPT1a, carnitine palmitoyltransferase 1a; GLUT, glucose transporter; MFP2, multifunctional protein‐2; SNAT1, sodium‐coupled neutral amino acid transporter 1. Created with BioRender.com.

Microglia, similar to other macrophages and immune cells, can utilize numerous fuel sources, and they possess the cellular machinery to potentially metabolize glucose, amino acids, and fatty acids, depending on the context (Bernier et al., 2020; Hammond et al., 2019; Yang et al., 2021) (Figure 1). Specifically, microglia express multiple glucose transporter isoforms, including GLUT3 and GLUT5 (which is specific to fructose) (Bernier et al., 2020; Payne et al., 1997), as well as transcripts for the proteins involved in glycolysis and oxidative phosphorylation (Bennett et al., 2016; Bernier et al., 2020; Ghosh et al., 2018; Monsorno et al., 2022), indicating that microglia utilize glucose metabolism for energy production. Glycolysis leads to the generation of pyruvate (and ATP) which then is converted to acetyl coenzyme A (Acetyl‐CoA) to begin the citric acid/Krebs cycle, through which FADH and NADH are produced and utilized to produce ATP by oxidative phosphorylation. Microglia also possess the transcripts for the glutamine transporter SNAT1 (Jin et al., 2015); glutamine can be converted to glutamate to participate in glutaminolysis, leading to ATP and NADH generation through a partial citric acid/Krebs cycle participation. Microglia further possess monocarboxylate transporters, which can transport lactate, pyruvate, and ketone bodies (Halestrap, 2013; Monsorno et al., 2022; Nijland et al., 2014). Lactate can be transformed to pyruvate primarily via lactate dehydrogenase B (Monsorno et al., 2022)—however, this can lead to the generation of reactive oxygen species (ROS) (Zhang et al., 2014); conversely, pyruvate can be metabolized to lactate to end glycolysis and shuttle lactate from oxidative phosphorylation, by lactate dehydrogenase A primarily (Monsorno et al., 2022). In rodents, lactate dehydrogenase B is expressed primarily in the CNS by microglia during early postnatal development, when there is high microglial heterogeneity and microglial‐dependent synaptic remodeling (Bennett et al., 2016; Mattei et al., 2020; Monsorno et al., 2022). Intriguingly, in oligodendrocytes, lactate shuttled from other cells may contribute to fatty acid synthesis and myelin production (Rinholm et al., 2011; Sánchez‐Abarca et al., 2001); although it remains to be seen if this also occurs in microglia.

Furthermore, microglia express the cluster of differentiation (CD)36, which may contribute to fatty acid uptake (Coraci et al., 2002). This was shown in N9 microglia‐like cells and human fetal microglial cells, as well as in post‐mortem brain samples of patients with Alzheimer's disease (AD) (Coraci et al., 2002). Also, *Cd36* transcript and CD36 protein levels were increased in myelin‐phagocytic microglia in mice in the context of demyelinating lesions indicating that the regulation of the expression of this fatty lipid translocase is important for myelin debris removal (Grajchen et al., 2020). However, whether CD36 leads to protective or detrimental microglial states is still a matter of debate and might depend on the method used to model demyelination and the time points evaluated (Grajchen et al., 2020; Hou et al., 2024). Since microglia possess CD36, those translocated fatty acids could be then on the route for mitochondrial fatty acid oxidation or peroxisomal fatty acid oxidation (Morito et al., 2024).

Microglia, at least under steady‐state conditions, may not possess the enzyme that transports fatty acids to mitochondria (CPT1a), which may indicate that fatty acid oxidation may not be a significant source of energy production, at least during normative conditions (Bernier et al., 2020; Jernberg et al., 2017). However, *Cpt1a* expression was found in mouse primary microglial cells and suppressed after different stimuli in vitro (i.e., interleukin (IL)1β/ interferon (IFN)γ or IL4) which indicates that microglia have the capacity to express *Cpt1a* under different conditions and thus oxidate fatty acids in mitochondria (Geric et al., 2019). Also, in vitro the CPT1 inhibitor etomoxir was shown to modulate gene expression of pro‐inflammatory cytokines in mouse primary microglial cells, providing metabolic evidence that CPT1a could modulate microglial states in vitro (Qin et al., 2023). Some work has demonstrated fatty acid oxidation also in microglia‐like BV‐2 cells (Bruce et al., 2018), but there is other evidence that these cells do not resemble microglia with other aspects of lipid metabolism (e.g., α/β‐hydrolase domain containing 6 activity) (Cao et al., 2019), complicating these results' interpretation.

Besides, regarding peroxisomal fatty acid oxidation, mouse primary microglial cells provided evidence that microglia express acyl‐CoA oxidase (ACOX), the rate‐limiting enzyme controlling the flux through the peroxisomal fatty acid oxidation pathway and multifunctional protein‐2 (MFP2), the enzyme responsible for hydration and second oxidation step in the process (Geric et al., 2019; Morito et al., 2024). Mice lacking *Mfp2*, specifically in microglia/macrophages, showed increased numbers of microglia and alterations in their morphology (shorter and thicker process) along with an increase in pro‐inflammatory cytokines (Beckers et al., 2019). Microglia from *Mfp2* knock‐out animals displayed a pro‐inflammatory profile compared to wild‐type animals under homeostatic conditions. Upon a pro‐inflammatory stimulus, microglia from *Mfp2* knock‐out animals were able to respond as microglia from wild‐type animals suggesting that peroxisomal fatty acid oxidation is required for microglial homeostatic conditions, but does not alter their inflammatory response (Beckers et al., 2019). Thus, the role of microglial fatty acid oxidation remains undetermined. This work taken together contributes to the understanding that microglia can utilize different energy sources. Currently, the field is investigating how the utilization of these energy sources is altered throughout development and into aging, as well as across disease states (Benarroch, 2022) (Figure 1). Furthermore, there is some evidence that the utilization of different energy substrates may lead to different microglial functions and this is a current major research focus in the field (Bernier et al., 2020).

As the brain is a lipid‐rich organ, it follows that microglia are intimately influenced by lipids. These cells have the capacity to both synthesize and respond to lipids. It is becoming progressively understood that microglial lipid composition and metabolic capacity are important for neural homeostasis and the regulation of other CNS cells (Folick et al., 2021). For example, microglia may contribute to fatty acid oxidation, contributing to brain energy levels, although as mentioned above, it is unclear if this occurs under steady‐state conditions (Bernier et al., 2020; Folick et al., 2021; Yang et al., 2021). This may change during disease conditions, for example, the in situ expression of enzymes associated with fatty acid oxidation was found in microglia in a mouse model of stroke (Loppi et al., 2023). Additionally, fatty acids may contribute to alterations in oxidative metabolism (Chausse et al., 2019; Loving & Bruce, 2020). Furthermore, alterations and dysregulation of microglial lipids, both their configuration and accumulation, are implicated across neurological diseases (Bruce et al., 2018; Nugent et al., 2020). In this review, we summarize the current understanding of lipids in microglia, including how this contributes to driving different microglial states and functions with consequences on brain health.

## 2 DIFFERENT MICROGLIAL STATES POINT TO LIPID PROCESSING VARIATIONS IN DEVELOPMENT AND DISEASE

Given microglia's diverse functions at steady state and throughout neurological conditions, it is important to understand their heterogeneity. In the past decade, single‐cell RNA‐sequencing (scRNA‐seq) and other emerging technologies have led to the discovery of numerous microglial transcriptional signatures and states (Paolicelli et al., 2022; Vecchiarelli & Tremblay, 2023b). In many cases, the identified microglial transcripts are related to lipid processing. For example, some transcripts altered in developmental signatures, including proliferative‐region‐associated microglia (PAM), are: *ApoE*, *Lpl*, and *Fabp5*. PAM and microglia with similar transcriptional signatures are found in white matter regions, such as the corpus callosum and white matter areas of the cerebellum (Li et al., 2019; Vecchiarelli & Tremblay, 2023b; Wlodarczyk et al., 2017). Some of these transcripts are also altered in microglia in different disease conditions. For example, disease‐associated microglia (DAM) and other microglial translational signatures associated with neurodegeneration and AD (in mice and humans) also show an increase in some of these lipid‐associated transcripts, including *APOE* and *LPL*, as do white matter associated microglia, which are found in the white matter of aged mice (Keren‐Shaul et al., 2017; Krasemann et al., 2017; Safaiyan et al., 2021; Sala Frigerio et al., 2019; Srinivasan et al., 2020; Vecchiarelli & Tremblay, 2023b). However, these microglial transcriptional signatures were identified via scRNA‐seq, which does not provide functional readouts (although it can inform future functional assays), nor does it provide information about lipids themselves. Additionally, using in situ approaches such as spatial transcriptomics and matrix‐assisted laser desorption/ionization (MALDI) will be important to undercover region‐specific changes in transcripts and lipid species, respectively.

### 2.1 Foamy and lipid droplet‐accumulating microglia

The accumulation of lipid granules/droplets in immune and glial cells (including putative microglia) has been described for over 100 years (Foley, 2010). Macrophages with irregular lipid accumulations have been of interest for the past 50 years; and in myeloid cells, it is well‐established that lipid droplets, which are cellular organelles that contain glycerolipids and cholesterols, are formed in response to inflammation and cellular stress (Bozza & Viola, 2010; den Brok et al., 2018), although the presence of lipid droplets does not necessarily indicate the presence of inflammation or cellular stress. For example, lipopolysaccharide (LPS), a pro‐inflammatory stimulus‐induced lipid droplet formation in N9 and BV‐2 microglia‐like cell lines and in vivo, in the hippocampus of young mice (Khatchadourian et al., 2012; Marschallinger et al., 2020). Lipid droplets are also important players in regulating cellular stress and help mouse primary microglial cells reduce their pro‐inflammatory profile after an LPS challenge (Li et al., 2023; Zadoorian et al., 2023). Lipid droplets can also be sites of production and storage of inflammatory mediators, including eicosanoids in leukocytes and pro‐inflammatory cytokines, such as IFN‐stimulated viperin in dendritic cells (den Brok et al., 2018; Saitoh et al., 2011). There have been numerous studies showing foamy microglia and lipid droplets in microglia ex vivo, in animal models and humans, including in the contexts of optic nerve injury, demyelination, excitotoxicity, hypoxia, encephalitis, and neurodegeneration (Arbaizar‐Rovirosa et al., 2023; Chali et al., 2019; Chiang et al., 1996; Dentinger et al., 1985; Derk et al., 2018; Dowding et al., 1991; Fabriek et al., 2005; Gedam et al., 2023; Kaur et al., 2002; Kodachi et al., 2017; Li et al., 1996; Ling, 1982; Liu & Shen, 1985; Luchetti et al., 2018; Machlovi et al., 2022; Sobaniec‐Lotowska, 2005; Sturrock, 1984; Tappe et al., 2019; Tremblay et al., 2016; van den Bosch et al., 2022; Victor et al., 2022; Wu et al., 2001; Xu et al., 2021). However, it is only in the last years that the field has investigated the potential functions of microglia with lipid accumulation.

**FIGURE 2:**
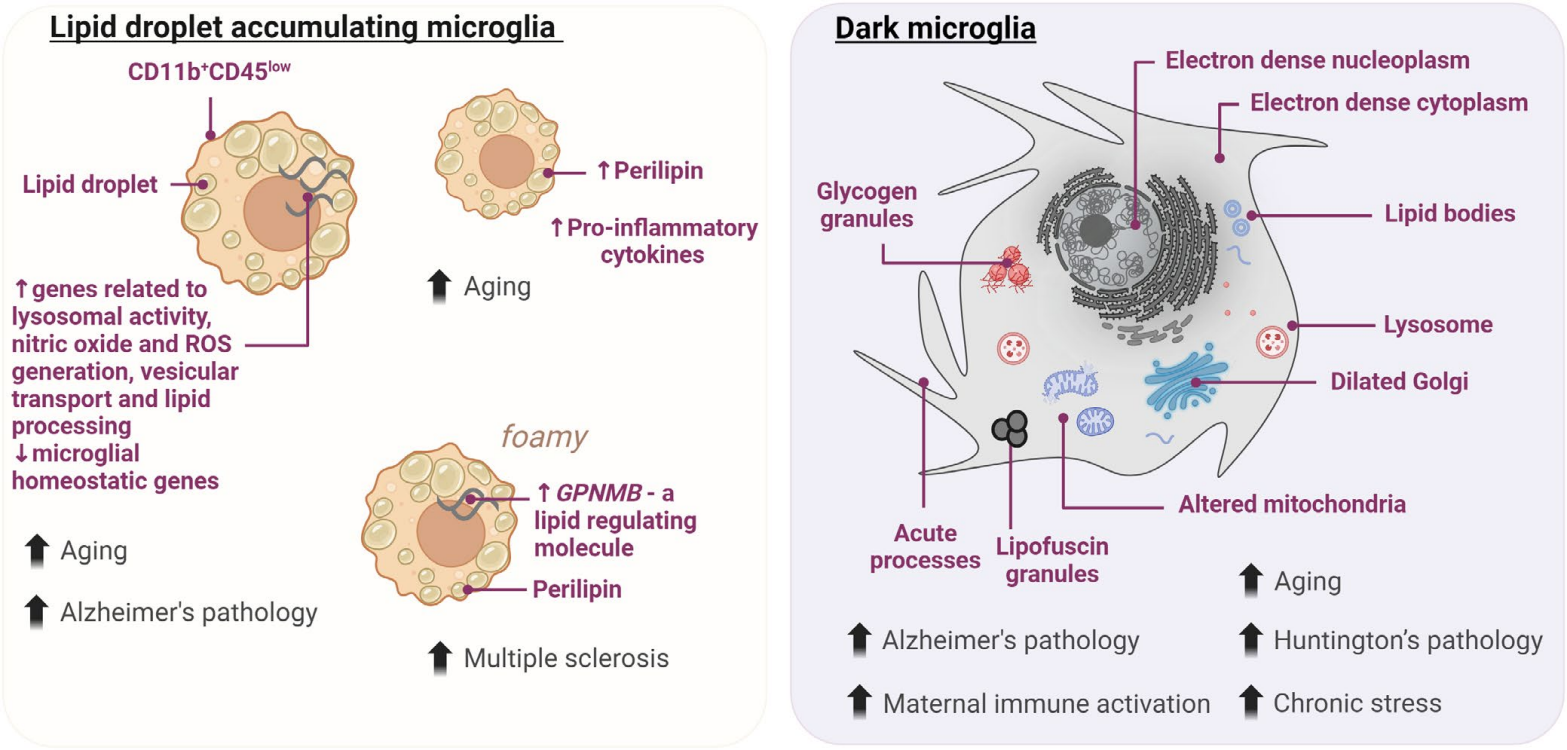
Features of lipid droplet accumulating and dark microglia. Different findings involving lipid droplet‐accumulating microglia and their association with aging and different pathological states. Ultrastructural characterization of dark microglia and in which models and pathologies they were found to be abundant. GPNMB, transmembrane glycoprotein NMB; ROS, reactive oxygen species. Created with BioRender.com.

Recently, a microglial transcriptional signature was identified in lipid droplet high CD11b^+^CD45^low^ cells from the hippocampus of adult mice, termed lipid droplet‐accumulating microglia (LDAM) (Marschallinger et al., 2020) (Figure 2). Microglia with lipid droplets were found via electron microscopy and fluorescence microscopy (with the lipid marker BODIPY) to be abundant in aged (20‐month‐old) male mice (and humans), but not in young male mice (3‐month‐old) (Marschallinger et al., 2020). The transcriptional signature associated with these cells included genes related to lysosomal activity, nitric oxide and ROS generation, vesicular transport, and lipid processing, but downregulation of microglial homeostatic genes (Marschallinger et al., 2020; Vecchiarelli & Tremblay, 2023b). In situ and in vivo these cells have an impaired phagocytic capacity (Marschallinger et al., 2020). Furthermore, these cells show increased ROS and inflammatory cytokine production, particularly in response to LPS stimulation (Marschallinger et al., 2020). Others have found similar LDAM in humanized mouse models of AD genetic risk‐associated pathology (Claes et al., 2021). An enrichment of LDAM microglia was reported in human post‐mortem brains from patients with AD, especially in those individuals with the *APOE4/4* genotype (Haney et al., 2024). These microglia expressed *ACSL1*, an enzyme involved in the first steps of lipid droplet biogenesis (Haney et al., 2024). Also, incubation of human‐induced pluripotent stem cell‐derived microglia with fibrillar Aβ induced the synthesis of triglycerides and accumulation of lipid droplets providing a link between hallmarks of AD pathology (Haney et al., 2024).

Single nucleus (sn)RNA‐seq also revealed transcriptional signatures in microglia isolated from post‐mortem samples of human patients with multiple sclerosis (Absinta et al., 2021; Vecchiarelli & Tremblay, 2023b). One such cluster of these ‘microglia inflamed in multiple sclerosis’ was termed foamy, and was associated with transcripts associated with lipid storage, lipoproteins, lysosomal activity, and inflammatory response, as well as a foamy morphology in situ (Absinta et al., 2021) (Figure 2). In a recent study combining in situ scRNA‐seq (MERFISH) and electron microscopy in adjacent brain sections, a foamy microglial state was observed following a demyelination injury in adult male mice; these cells upregulated a number of transcripts, including for transmembrane glycoprotein NMB (GPNMB) (a lipid regulating molecule) and those associated with the lysosome as well as cholesterol and lipid metabolism (Androvic et al., 2023). Intriguingly, progranulin (*Grn*) knock‐out increased the numbers of LDAM, including in white matter regions (Marschallinger et al., 2020). Progranulin also regulates GPNMB (Houser et al., 2022), which is found in PAM (localized to white matter regions during development) (Li et al., 2019). It was also reported that in a zebrafish injury model, the loss of *Grn* led to prolonged microglial reactivity (including an increase in lipid droplet accumulation), impaired neurogenesis, and reduced microglial flexibility—preventing a return to homeostatic microglia following injury (Zambusi et al., 2022). Recent work has highlighted that a loss of progranulin led to the accumulation of lipid droplets in microglia in the corpus collosum of male mice with a demyelination injury model, but not female mice, highlighting potential sex differences in this phenomenon (Zhang et al., 2023).

In aged female mice (18–22‐month‐old), microglia similarly accumulated lipids, while these cells had increased levels of perilipin, a molecule that is associated with the production of lipid droplets; furthermore, perilipin was associated with the production of the pro‐inflammatory cytokine, tumor necrosis factor (TNF) (Shimabukuro et al., 2016). In a mouse model of toxin‐induced demyelination, perilipin‐2 protected lipid droplets from lipolysis‐mediated degradation—therefore, a loss of perilipin‐2 leads to an increased turnover of lipid droplets in foamy microglia as well as improves remyelination in this injury model (Loix et al., 2022).

Alterations in lipid droplets in microglia point to potential dysregulation of microglial lipid metabolism. In primary microglial cells from male rat pups, lipid droplet accumulation was abrogated with 48 h of glucose deprivation, and this perhaps indicates that microglia switch to utilizing lipid energy stores, reducing lipid accumulation during conditions of reduced glucose (Churchward et al., 2018). Antagonism of the leukotriene receptor in a mouse model of AD pathology had reduced expression of lipid droplet‐accumulation‐related microglial transcripts, indicating a potential role for arachidonic acid derivatives in regulating lipid droplet formation (Michael et al., 2021). In male and female adult mice, the dark (active) phase of the light cycle was associated with an increased expression in microglia of uncoupling protein 2, a mitochondrial transporter protein that regulates ROS production, following synaptic remodeling (increased synapse phagocytosis) in the light phase; knock‐out of this protein in CX3CR1+ microglia cell led to impairment of this light phase associated reduction of synapses and further led to increases in ROS and lipid droplets (Yasumoto et al., 2021). Interestingly, microglial knock‐out of uncoupling protein 2 also led to altered hippocampal circuit electrophysiology and increased anxiety‐like behavior (in males); this may indicate that there is a normative accumulation of lipid droplets for the clearance of phagocytosed elements, but in conditions of disease or aging, there is impairment of this process (Yasumoto et al., 2021).

Triggering receptor expressed on myeloid cells 2 (TREM2), a receptor for lipids expressed by microglia, is important for their phagocytic capacity, immune responses, and lipid metabolism (Gouna et al., 2021). Knock‐out of *Trem2* prevented the formation of foamy microglia with lipid droplets in an acute toxin‐induced demyelination model, which was associated with impaired remyelination (Gouna et al., 2021). Furthermore, in induced pluripotent stem cell (iPSC)‐derived microglia from patients with Nasu‐Hakola disease, which is caused by mutations in *TREM2*, there were reduced lipid droplet numbers (Filipello et al., 2023). This is additional evidence that lipid droplets may be useful to buffer excess lipids, particularly in the acute injury phase, creating an environment for regeneration, but that may in some contexts or with chronic accumulation, this buffering capacity being overwhelmed, perhaps, leading to impaired beneficial functions of microglia.

The use of complementary in vivo technologies will allow for further examination of the function of these cells. For example, in rats exposed to a hypoxia/stroke model (medial cerebral artery occlusion), fatty acyl proton signals detected by magnetic resonance spectroscopy were positively correlated with lipid droplets in OX42+ cells (microglia/macrophages) (Gasparovic et al., 2001).

### 2.2 Dark microglia

Dark microglia are a particular microglial state described using high‐spatial‐resolution electron microscopy (reviewed in St‐Pierre et al. (2020)) (Figure 2). They were named after their dark appearance because of their electron‐dense cytoplasm and nucleoplasm, a feature that could be related to oxidative stress (Bisht et al., 2016). Indeed, dark microglia display alterations in organelles related to cellular stress, including Golgi apparatus and endoplasmic reticulum dilation as well as altered mitochondria, characterized by a deterioration of the outer membrane, degradation of the cristae or “holey” shape (i.e., mitochondria forming a donut shape) (St‐Pierre et al., 2020; St‐Pierre et al., 2022). Interestingly, this microglial state has been found in pathological conditions, such as mice exposed to maternal immune activation and rodent models of AD pathology, Huntington's disease pathology and amyotrophic lateral sclerosis, but it is nearly absent in the brain of healthy adult mice (El Hajj et al., 2019; Garofalo et al., 2022; Guma et al., 2022; Hui et al., 2018; Savage et al., 2020). Also, dark microglia were reported in aging human post‐mortem brain samples indicating that dark microglia also occur in humans (St‐Pierre et al., 2022).

Some of the features described by dark microglia suggest that they have a unique metabolic profile: along with the mitochondrial alteration they show an accumulation of glycogen granules (St‐Pierre et al., 2022). These glycogen granules were found in microglial cells located near amyloid beta (Aβ) plaques and dystrophic neurites in the hippocampus of aged APP‐PS1 mice, a model of AD pathology. Even when both typical and dark microglia near the plaques and dystrophic neurites showed ultrastructural evidence of glycogen granules in the cytoplasm, they were much more frequent in dark than typical microglia (St‐Pierre et al., 2022). Similarly, increased glycogen accumulation characterizes macrophages/microglia in aged mice and humans (Minhas et al., 2021). Human monocyte‐derived macrophages from individuals over 65 years old showed an enhanced prostaglandin E2—a lipid messenger—signaling through the EP2 receptor (Minhas et al., 2021). This led to the activation of glycogen synthase and subsequent glycogen accumulation, reducing the glucose flux (Minhas et al., 2021). This is particularly important since macrophages from aged individuals lose their ability to use different substrates aside from glucose to obtain energy (Minhas et al., 2021). In the mouse hippocampus, EP2 protein expression was restricted to IBA1+ cells (microglia/macrophages) and pharmacological inhibition of EP2 reduced CD68 levels, glycogen synthesis, and enhanced glycolysis and the Krebs' cycle, as well as restored the ultrastructure of mitochondria in microglia (Minhas et al., 2021). These findings support the idea that metabolic shifts in microglia could be under control of lipid messengers. The metabolic pathways that characterize dark microglia fuel usage and how this impacts their function are under investigation.

Dark microglia processes also show some particular features that differentiate them from those of other microglial states as they are thin and form numerous acute angles (Bisht et al., 2016). They directly contact blood vessels, ensheathing the basement membrane, thus indicating their contribution to the glia limitans and the neurovascular unit (Bisht et al., 2016). Also, they extensively encircle axon terminals and dendritic spines suggesting that they could be involved in synaptic pruning and plasticity (Bisht et al., 2016). What signals lead to this microglial state and what their functional outcome is still a matter of research.

## 3 LIPID SIGNALING IN MICROGLIA

Different microglial transcriptional signatures and states are associated with altered lipid processing, as described previously (Vecchiarelli & Tremblay, 2023b) and above. In the following section, we discuss some commonly altered lipid pathways, using those that are altered in these states as a guide.

### 3.1 Lipoprotein lipase

**FIGURE 3:**
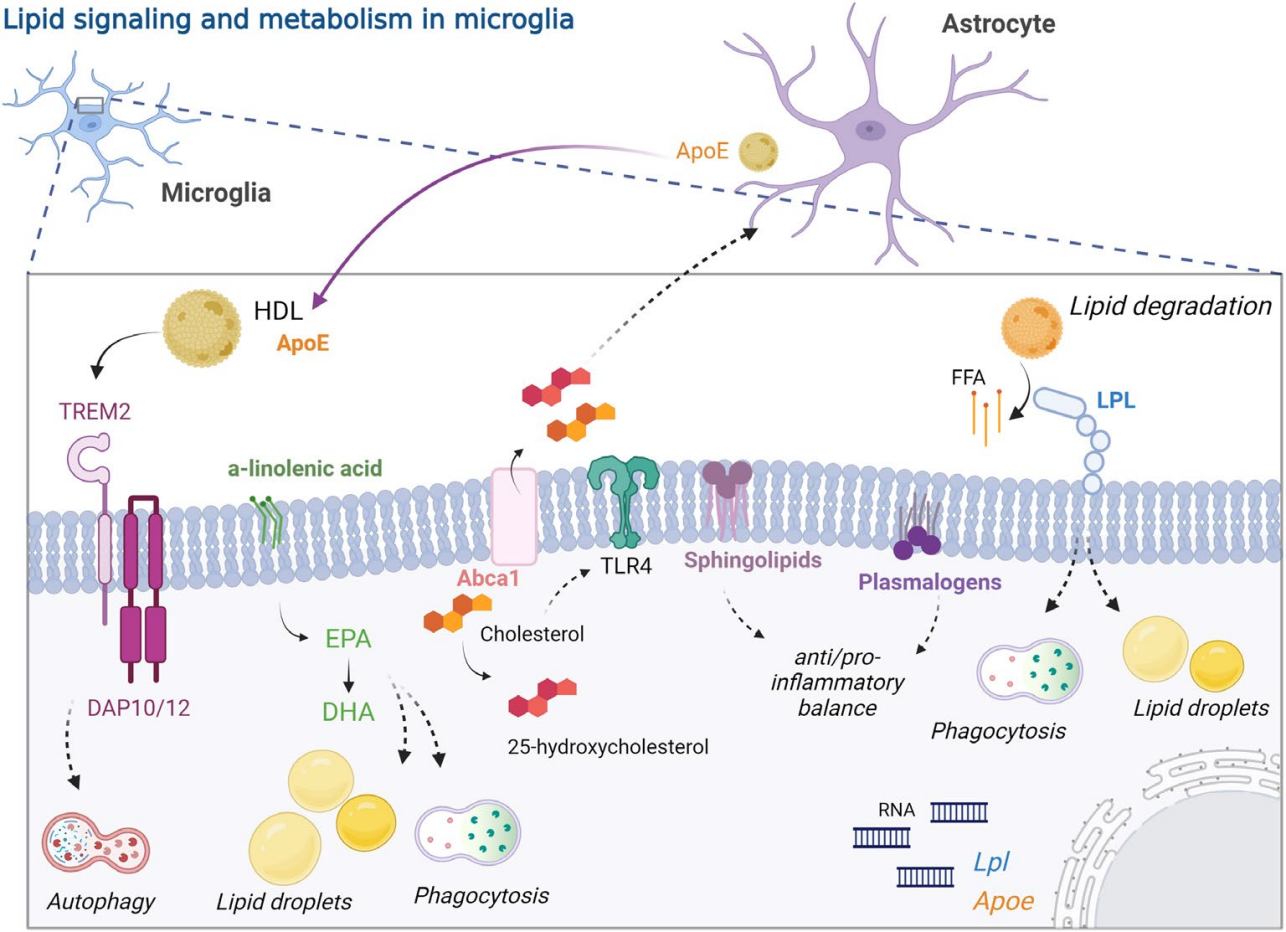
Lipid signaling and metabolism in microglia. Astrocytes secrete lipidated APOE which is recognized in turn by microglial TREM2, a pathway related to disease‐associated microglia transformation and autophagy. Omega‐3 (a‐linolenic acid) and its derivatives shape lipid droplet size and number and phagocytic activity in microglia. Cholesterol derivatives determine astrocytic metabolism and pro‐inflammatory microglial response. Sphingolipids and plasmalogens are involved in determining the pro‐ and anti‐inflammatory balance in microglia. LPL degrades lipids and it is tightly associated with lipid droplet accumulation and phagocytic activity in microglia. ABCA1, ATP binding cassette subfamily A member 1, APOE, apolipoprotein E; DHA, docosahexaenoic; EPA, eicosapentaenoic; FFA, free fatty acids; HDL, discoidal high‐density lipid; LPL, lipoprotein lipase; RNA, ribonucleic acid; TLR4, Toll‐like receptor 4; TREM2, triggering receptor expressed on myeloid cells 2. Created with BioRender.com.

Lipoprotein lipase (LPL) is an extracellular enzyme that hydrolyses triglycerides into non‐esterified fatty acids and monoacylglycerol (Mead et al., 2002) (Figure 3). It is highly expressed in adipose tissue, cardiac and skeletal muscles, and the lactating mammary gland (Mead et al., 2002). Its catalytic function enables posterior lipid storage in the white adipose tissue or storage/oxidation in muscular cells (Mead et al., 2002). In addition to their metabolic role in the periphery, non‐catalytic functions have also been described and are still under study, particularly in the CNS (Wang & Eckel, 2012).

The analysis of *Lpl* expression throughout the CNS gave the first insight that *Lpl* was expressed in defined regions and not only associated with the vasculature (Bessesen et al., 1993). In the adult rat brain, *Lpl* mRNA expression evaluated by in situ hybridization and coincident LPL protein expression assessed by immunohistochemistry were reported in neuronal cell bodies of the hippocampus and Purkinje cell layer of the cerebellum (Bessesen et al., 1993). A subsequent study that evaluated *Lpl* mRNA expression by in situ hybridization confirmed that *Lpl* was indeed expressed in several brain areas with the highest expression found in the pyramidal cell layers of the hippocampus in adult mice (Paradis et al., 2004). Interestingly, *Lpl* mRNA levels were increased in the penumbral area of an ischemic lesion in mice and colocalized with IBA1+ cells (Paradis et al., 2004). This suggests that *Lpl* expression can be induced in pathological contexts, particularly in phagocytic cells. Indeed, a particular microglial state, DAM, that upregulates *LPL/Lpl* expression was found near Aβ plaques both in the 5xFAD mouse model of AD pathology and in post‐mortem brain tissue from patients with AD (Keren‐Shaul et al., 2017). Interestingly, dark microglia have also been found in the vicinity of plaques containing fibrillar materials and encircling dystrophic neurites and synaptic elements in AD pathology mouse model (APPSwe‐PS1Δe9) and post‐mortem brain samples from patients with AD suggesting that these cells could be involved in active phagocytosis (El Hajj et al., 2019; St‐Pierre et al., 2022). Coincidently, treatment of BV‐2 cells or rat primary microglial cultures with Aβ induced the expression of LPL. In this context, *Lpl* silencing with short hairpin (sh)RNA in BV‐2 cells reduced Aβ phagocytosis, linking the expression of LPL to microglial phagocytic performance (Ma et al., 2013).

During early postnatal development in rats, the activity of LPL in the whole brain increases reaching a peak between postnatal day (P) 5 and P10 (Nuñez et al., 1995). The hippocampus shows the most remarkable increase (Nuñez et al., 1995). The hippocampus also remains the structure with the highest enzymatic activity in adulthood, coincident with LPL expression (Bessesen et al., 1993; Nuñez et al., 1995). Its temporal pattern of activity coincides with the onset of the myelination process during development (Zeiss, 2021). Considering the need of lipids for this process, LPL arises as a promising key player in myelination (Nuñez et al., 1995; Zeiss, 2021).

Of note, PAM upregulated genes involved in lipid processing including *Lpl*, *Fabp5*, and *Apoe* at P7 in mice (Li et al., 2019). Supporting the hypothesis of PAM's involvement in myelination was that their main location in developing white matter (Li et al., 2019). This microglial state was shown to be involved in active phagocytosis of oligodendrocytes and astrocytes during development (Li et al., 2019). Although studies regarding LPL function during development are scarce, recent studies associate LPL with the remyelination process, especially associated with microglial functions. In mice with experimental autoimmune encephalitis as a model of demyelinating disease in humans, the activity of LPL was increased after 30 days coincident with initiation of symptom improvement (Bruce et al., 2018). Using an in vitro approach, the authors showed that treatment of BV‐2 cells with an *Lpl* shRNA lentiviral vector to reduce LPL expression induced a pro‐inflammatory state and their secretome impeded remyelination in brain slices treated with lysolecithin, a demyelinating agent (Bruce et al., 2018). These findings suggest that LPL expression is necessary for a microglial repair phenotype, and indeed BV‐2 LPL‐expressing cells showed increased internalization of lipids in vitro (Bruce et al., 2018). In fact, the reduced expression of LPL led to the accumulation of lipid droplets, along with a diminished lipid uptake and fatty acid oxidation, suggesting that LPL is a key regulator of lipid storage and usage (Loving et al., 2021). In post‐mortem brain samples of patients with multiple sclerosis, LPL immunostaining was increased close to active demyelinating lesions and colocalized with IBA1+ cells (Kamermans et al., 2019). Treatment of macrophages in vitro with antagonists of LPL activity reduced lipid uptake but did not affect myelin phagocytosis suggesting that other mechanisms could be involved in lipid metabolism of myelin by macrophage and microglia (Kamermans et al., 2019).

Overall, microglial expression of LPL seems to be tightly regulated and is among the set of genes that characterize different microglial states' transcriptomic signatures (i.e., PAM and DAM). This suggests that LPL is involved in key processes during development and pathology, with myelination and its relationship to microglial phagocytic activity being the most promising link to focus on.

### 3.2 Apolipoprotein E

APOE is mostly expressed by astrocytes in the CNS under homeostatic conditions (Xu et al., 2006). This apolipoprotein is secreted and lipidated with cholesterol and phospholipids forming discoidal high‐density lipid (HDL) particles and reducing the lipid content within the cells (reviewed in Wolfe et al. (2019)) (Figure 3). These lipids can be then redistributed to other cells, making APOE a main regulator of cholesterol distribution within the CNS. In the case of microglia, even under conditions such as a kainic acid insult in mice, their expression of APOE was restricted to only 6 ± 3% of CD11b+ cells (Xu et al., 2006). Recently, using scRNA‐seq, *ApoE* was found to be upregulated in the early transition from homeostatic microglia to DAM in the 5xFAD mouse model of AD pathology (Keren‐Shaul et al., 2017). Overall, *ApoE* is upregulated by and characterizes a subset of microglia under pathological conditions.

APOE displays three different allelic variants in humans, which leads to different isoforms named *APOE2*, ‐*E3*, and ‐*E4*, with *APOE4* being the major genetic risk factor for AD. A recent study addressed the relationship between this isoform and 18‐kDa translocator protein (TSPO) expression in the brain. The study, which included 79 healthy controls, 23 participants with mild cognitive impairment, and 16 participants with AD dementia, showed using positron emission tomography that [^11^C]PBR28‐TSPO uptake was higher in *APOE4* carriers in cortical areas such as the transentorhinal and entorhinal cortices, as well as the hippocampus, and this was further associated to cognitive decline and hippocampal atrophy (Ferrari‐Souza et al., 2023). These results could be interpreted as overall “neuroinflammation,” but altered neuronal activity can increase TSPO and thus can lead to a mistaken interpretation (Notter et al., 2021). However, in support that microglial responses could be associated with APOE isoforms, it was observed that microglia stained for IBA1 were less ramified in patients with dementia, particularly in the hippocampus and superior and middle temporal gyrus. *APOE4* carriers with dementia further showed an increase of rod microglia in the superior and middle temporal gyrus (Kloske et al., 2023). Rod microglia is an elongated microglial morphology with two long primary processes but scarce secondary ones, which has been consistently reported in preclinical models and post‐mortem samples of patients with different pathologies, including AD and epilepsy (reviewed in Giordano et al. (2021)). Also, patients with AD who carry *APOE4* showed an increase in perilipin‐2 colocalizing with IBA1+ cells and NeuN+ cells, suggesting that there is an increase in lipid droplets in both microglia and neurons, respectively (Wang et al., 2023).

In rodents, APOE4 was shown to regulate microglial metabolism in aged mice (Lee et al., 2023). A subset of microglial cells obtained from aged mice expressing human *APOE4* showed an increase in *Apoe4* expression along with upregulation of genes related to glycerolipid metabolism and glycolysis. Of note, this subset was also characterized by genes associated with the DAM/microglial neurodegenerative phenotype (MGnD) signatures, including *Lpl*, *Ch25h*, *Fabp5*, and *Apoe* (Lee et al., 2023). When addressing these cells' metabolism through a Seahorse platform, E4 microglia showed enhanced glycolysis and reduced mitochondrial respiration compared to E3 microglia, and when exposed to an inflammatory stimulus as LPS, they increased further the glycolytic ATP production whereas E3 microglia were able to increase mitochondrial respiration (Lee et al., 2023). In a cuprizone model of demyelination, microglia in *Apoe4* mice showed an accumulation of lipid droplets and decreased phagocytic activity suggested by a decrease in CD68 staining compared to *Apoe2* mice, which rendered impaired remyelination (Wang et al., 2022). These results indicate that APOE isoforms can shape microglial metabolism and phagocytic function.

APOE can interact with low‐density lipoprotein receptor (LDLR) and LDLR‐related receptor 1 (LRP1), as well as with TREM2 which is expressed in myeloid cells including microglia (reviewed in Wolfe et al. (2019)). Indeed, TREM2 and APOE signaling are tightly linked to the microglial state. Apoptotic neurons are able to activate this cascade transforming microglia from homeostatic to MGnD (Krasemann et al., 2017). Also, TREM2 proved to be critical for microglial metabolism in the 5xFAD model of AD. The absence of *Trem2* leads to an increase in autophagy along with a derailment of the mTOR pathway in microglia (Ulland et al., 2017). In line with these findings, the absence of *Trem2* in APPSwe‐PS1Δe9 mice led to an increase in cognitive impairment, and reduced recruitment of microglia to the plaques and larger plaques (Fitz et al., 2020). Mounting evidence suggests that the APOE‐TREM2 pathway is crucial to determine the microglial state during AD pathology. In contrast with these findings, during postnatal development, even though PAM share similarities to the DAM transcriptional signature, they do not seem to depend on APOE‐TREM2, suggesting that other mechanisms could be involved during development (Li et al., 2019). Microglial TREM2 also plays a protective role in stroke (Wei et al., 2024). TREM2, as well as TGFβ1, was upregulated in mouse primary microglial cells under oxygen–glucose‐deprived conditions (Wei et al., 2024). By silencing *Trem2*, microglia accumulated lipid droplets and decreased TGFβ1 expression (Wei et al., 2024). This was also proved in vivo, where knocking down *Trem2* resulted in increased cerebral infarct size and worsened behavioral outcomes (Wei et al., 2024).

### 3.3 Polyunsaturated fatty acids (focus on n‐3 PUFA)

There are two primary families of polyunsaturated fatty acids (PUFA), n‐6/omega‐6 and n‐3/omega‐3, the prototypical of which being, respectively, linoleic acid—the precursor for arachidonic acid—and α‐linolenic acid—the precursor of eicosapentaenoic (EPA) and docosahexaenoic (DHA) acids (Layé et al., 2018) (Figure 3). Linoleic acid and α‐linolenic acid are essential fatty acids obtained solely from diet/consumption (Layé et al., 2018). Recent work indicates that microglia are enriched in EPA (Cisbani et al., 2021). While n‐3 PUFA was previously considered to be inert components of cellular membranes, research from the past two decades has focused on ascertaining a role for these molecules in microglial function.

N‐3 PUFA supplementation or deprivation alters lipid profiles in microglia. For example, n‐3 PUFA‐enriched diets given to mouse dams increased EPA and phosphatidylinositol, and phosphatidylserine levels in microglia isolated from pups (Rey et al., 2018). Deficiency of n‐3 PUFA throughout gestation and lactation in mice changed the lipid composition in the pups' brain, decreasing n‐3 PUFA and increasing n‐6 PUFA (Madore et al., 2014). In the hippocampus, a deficient n‐3 PUFA diet leads to a reduction in microglial process motility as well as slower processes retraction (Madore et al., 2014). Also, microglia from n‐3 PUFA‐deficient animals showed an altered bioactive lipids profile with greater levels of arachidonic acid‐derived mediators and lower amounts of DHA‐ and EPA‐derived mediators (Madore et al., 2020). Boosting n‐3 PUFA levels, whether through dietary or genetic means, has been shown to reduce microglial numbers, both in the steady state and in response to disease or injury states (Baazm et al., 2021; Dinel et al., 2016; Hakimian et al., 2019; Hopperton et al., 2016; Jiang et al., 2016; Kalogerou et al., 2022; Mondal et al., 2021; Tenorio‐Lopes et al., 2017). Conversely, reducing DHA levels increased microglial numbers (Talamonti et al., 2020). This also occurred in mouse pups when their dams were deprived of n‐3 PUFA; furthermore, microglia from pups of dams with n‐3 PUFA deficiency had increased dendritic spine phagocytosis, showing increased cellular inclusions (including spines) and increased post‐synaptic density‐95 levels (Madore et al., 2020). However, the outcomes of n‐3 PUFA on the microglial number may be dependent on age and brain region, as DHA deficiency in dams reduced microglia number, as well as contact with myelin, in pups at P10 in white matter regions, as well as increased microglial mitochondria number and cell proportion (Decoeur et al., 2020). This corresponds to postnatal time points when there is high microglial heterogeneity and the emergence of microglial states, such as PAM or dark microglia (Vecchiarelli & Tremblay, 2023b). This may indicate that n‐3 PUFA changes in microglia may be particularly important for altering microglial non‐homeostatic microglia states in development. Furthermore, there are sex differences in the effects of DHA deficiency on microglia, as in females, DHA deficiency has been shown to reduce microglial number (Rodríguez‐Iglesias et al., 2022). Together this highlights a need for increased specificity using more in vivo analysis of the effects of fatty acid supplementation and depletion on microglia, paying careful attention to region, duration, time point, and sex.

There are a number of reports showing that n‐3 PUFA exposure can alter microglial inflammatory signaling. Exposure to n‐3 PUFA prevented LPS‐induced increases in matrix metalloproteinase (MMP)9, an enzyme involved in the degradation of the extracellular matrix, in rat primary microglial cells (Liuzzi et al., 2007). Exposure to n‐3 PUFA EPA and DHA in primary microglial cells reduced nitric oxide in response to LPS, as well as, to IFNγ and myelin (Chen et al., 2014). N‐3 PUFA administration also reduced levels of CD16+ IBA1+ microglia but increased CD206+ IBA1+ microglia in the cortex following traumatic brain injury in adult male rats, potentially through reducing HMGB1/NF‐kB pathway signaling (Chen et al., 2018). DHA promoted small lipid droplet formation and normalized LPS‐induced large lipid droplet formation in microglia from organotypic hippocampal slices from P6‐P8 mice (Chang et al., 2015). In rat primary microglial cells, DHA administration blunted Japanese Encephalitis Virus infection induced increases in pro‐inflammatory cytokines (IL‐1β and ‐6, and TNF) (Chang et al., 2021). Additionally, n‐3 PUFA impacts microglial phagocytic activity. Incubation of mouse primary microglial cells with arachidonic acid increased the phagocytosis of synaptosomes in vitro while incubation with DHA or EPA decreased their phagocytic activity (Madore et al., 2020). This increase in phagocytosis was partially mediated by 12/15‐lipoxygenase (LOX)/12‐HETE signaling (Madore et al., 2020), supporting the idea that bioactive lipidic mediators play a key role in modulating microglial functions. In contrast to the phagocytosis of synaptosomes, DHA and EPA led to an increased phagocytosis of myelin by mouse primary microglial cells (Chen et al., 2014), suggesting that their effect on phagocytic activity depends on the stimuli and context.

Together these works reveal an emerging role for n‐3 PUFA in regulating microglial form and function. As n‐3 PUFA can be supplemented in the diet, the study of their function is increasingly important, as both an understanding of the effects of this common dietary supplementation, as well n‐3 PUFA usage as potential therapeutic agents (Layé et al., 2018).

### 3.4 Cholesterol

Cholesterol is abundant in the brain—this organ contains around 20% of all whole‐body cholesterol content—and most of it (up to 80%) is localized in myelin sheaths (Yutuc et al., 2020; Zhang & Liu, 2015). Cholesterol is a key component of cellular membranes and serves as a precursor for multiple active metabolites. Since blood‐circulating lipoproteins are prevented from crossing the brain–blood barrier, cholesterol synthesis in the brain occurs de novo. In fact, there is a burst of cholesterol synthesis that occurs during developmental myelination and after this process, cholesterol turnover becomes low and steady through adulthood (Zhang & Liu, 2015). Microglial cholesterol metabolism is currently gaining attention, particularly for its role in regulating pro‐ and anti‐inflammatory states, as well as their relation to the remyelination process. As myelin sheaths are enriched in cholesterol, it is interesting to consider how microglia deal with phagocyting myelin as it could give a hint on potential therapeutic targets for demyelinating diseases.

Mouse primary microglial cells exposed to LPS in vitro secreted 25‐hydroxycholesterol, a cholesterol metabolite, that was internalized by astrocytes, suggesting that this metabolite could be involved in cell–cell communication in a pro‐inflammatory context (Cashikar et al., 2023) (Figure 3). Indeed, it induced the secretion of APOE containing cholesterol in mouse primary astrocytes, facilitating cholesterol distribution among cells and possibly influencing the function of other cells including microglia (Cashikar et al., 2023). For example, the presence of APOE in mouse primary microglial cells enhanced the efficiency of Aβ degradation by reducing the intracellular levels of cholesterol (Lee et al., 2012). Also, the presence of 25‐hydroxycholesterol regulated the expression of key genes involved in cholesterol transport—upregulation of *Abca1* and downregulation of *Ldlr*—and biosynthesis—downregulation of *Srebf2*, *Insig1*, *Acat2*, *Hmgcr* and *Dhcr24*—which resulted in increased cholesterol efflux and reduced biosynthesis in astrocytes (Cashikar et al., 2023). All these changes resulted in a diminished intracellular free cholesterol content together with an increase in intracellular cholesteryl esters stored in lipid droplets in astrocytes (Cashikar et al., 2023). Also, exposure to 25‐hydroxycholesterol mimicked the LPS‐detrimental effects on long‐term potentiation in rat hippocampal slices (Izumi et al., 2021). Since knock‐out mice for *Ch25h*—a gene that encodes an enzyme required for the synthesis of 25‐hydroxycholesterol—did not show these alterations, it is postulated that this cholesterol metabolite is required for the detrimental effects of LPS (Izumi et al., 2021). Additionally, primary microglial cells obtained from *Ch25h* knock‐out mice showed a reduction of secreted IL‐1β after LPS exposure, suggesting that this metabolite is also involved in specifically pro‐inflammatory cytokines release. Primary microglial cells obtained from mice expressing human *APOE4* produced higher amounts of 25‐hydroxycholesterol and this was associated with an increase in IL‐1β secretion following LPS challenge compared with microglial cells obtained from mice expressing *ApoE2‐* or *ApoE3‐* (Wong et al., 2020). In line with these findings, it was reported that an injection of 25‐hydroxycholesterol into the corpus callosum of mice induces the recruitment of IBA1+ cells and increased levels of IL‐1β (Jang et al., 2016). However, another study showed anti‐inflammatory properties of 25‐hydroxycholesterol, as it reduced IFNγ signaling in mouse primary microglial cells by disrupting lipid rafts and impeding the increase of caveolin‐1 necessary for the internalization by endosomes and subsequent signaling by IFNγ (Lee et al., 2022). This resulted in a reduction of neurotoxic effects of mouse primary microglial cells when co‐cultured with mouse primary neuronal cells (Lee et al., 2022). These opposing effects of 25‐hydroxycholesterol could be because of different concentrations used across studies, with low concentrations more associated with an anti‐inflammatory effect and higher concentrations with a pro‐inflammatory response (Jang et al., 2016; Lee et al., 2022; Wong et al., 2020).

Cholesterol metabolism is also implicated in pain regulation. Cisplatin, a platinum‐based alkylating agent used in chemotherapy, caused tactile allodynia in mice and spinal microglia of these animals showed an increase in lipid raft formation. This could lead to a hyperactivation of Toll‐like receptor (TLR)4, since dimerization of TLR4 which occurs in lipid rafts is the first step of TLR4 activation (Navia‐Pelaez et al., 2021). Bulk RNA‐sequencing showed that CD11b+ TMEM119+ FACS‐sorted spinal microglia from cisplatin‐treated mice down‐regulated cholesterol transporters *Abca1* and *Abcg1*, lysosomal related genes and upregulated arachidonic acid metabolism genes (Navia‐Pelaez et al., 2021). Spinal microglia from animals treated with cisplatin shared an RNA expression profile with DAM, as well as an increased number and size of lipid droplets (Navia‐Pelaez et al., 2021). When *Abca1* and *Abcg1* were knocked down in microglia, animals showed allodynia in the absence of stimuli along with higher TLR4 expression and dimerization as well as lipid raft content in spinal microglial cells compared with wild‐types (Navia‐Pelaez et al., 2021). These data support the idea that cholesterol transport in microglia is tightly related to their immune response and function.

Another example is Niemann‐Pick type C (NPC) disease, a rare lipid storage disorder in which most of the patients carry mutations in the *NPC1* gene (Colombo et al., 2021). This gene encodes for a protein involved in the transport of lipids from late endosomes/lysosomes to other organelles, so when this route is impaired, lipids accumulate in the endosomes/lysosomes. Microglia express *NPC1* and microglia from animals lacking *Npc1* showed an increase in intracellular cholesterol which colocalized with CD68, suggesting that the cholesterol was accumulated in endosomes/lysosomes (Colombo et al., 2021). When analyzing their proteomic signature, microglia showed a similar signature to DAM, downregulating homeostatic proteins and upregulating TREM2, TYROBP, APOE, ITGAX/CD11c, and many late endosomal/lysosomal proteins, including LAMP1/2, LIPA, CD68, CTSB, CTSD, and GRN (Colombo et al., 2021). This shows that the phagolysosomal pathway is strongly upregulated even when lipid flux is impeded. Indeed, microglia obtained from *Npc1* knock‐out mice showed an increase in phagocytic activity. This preceded neuronal loss in this animal model. Also, myelin phagocytosis was enhanced but myelin turnover and recycling were impaired leading to an intracellular accumulation of myelin in the late endosomal/lysosomal compartment of microglia. This was in contrast to what happened in wild‐type microglia, where phagocytosed myelin was stored in lipid droplets. Indeed, microglial cholesterol regulation is key for remyelination in the context of demyelinating diseases, such as multiple sclerosis (Berghoff et al., 2022). Under basal conditions, microglia did not express high levels of sterol genes but under acute demyelination (mice treated with cuprizone) they upregulated these genes (Berghoff et al., 2021). Mice carrying mutations both in sterol synthesis (squalene synthase) or cholesterol efflux (*Abca1* and *Acbg1*) genes specifically in CX3CR1+ microglia also demonstrated that both processes are needed for restoration of myelin after acute demyelination induced by cuprizone treatment (Berghoff et al., 2021). These mutant mice showed foamy microglia suggesting a compromised lipid turnover and recycling (Berghoff et al., 2021). In particular sterol synthesis at the level of squalene synthase was critical to switch a microglial pro‐inflammatory state towards one that enables remyelination (Berghoff et al., 2021). This was possible through the accumulation of desmosterol and activation of liver X receptor (LXR)‐signaling, which led to increased cholesterol efflux and recycling (Berghoff et al., 2021). Another study showed that stearoyl‐CoA desaturase‐1, the rate‐limiting enzyme in the desaturation of saturated fatty acids, was responsible for perpetuating macrophage/microglial pro‐inflammatory state, thus leading to free cholesterol accumulation because of inhibition of ABCA1‐mediated cholesterol efflux (Bogie et al., 2020). Inhibition of stearoyl‐CoA desaturase‐1 also resulted in an improvement in remyelination in ex vivo cerebellar brain slices demyelinated with lysolecithin and in vivo in the cuprizone‐induced de‐ and remyelination model (mice treated with cuprizone) (Bogie et al., 2020). TREM2 was additionally found to control lipid metabolism in the chronic demyelination induced by cuprizone (mice fed with cuprizone for 12 weeks) (Nugent et al., 2020). After demyelination induced by cuprizone, microglia upregulated *Trem2*‐dependent genes involved in cholesterol transport and metabolism, including *Apoe*, *Apoc1*, *Ch25h*, *Lipa*, *Nceh1*, *Npc2*, and *Soat1* (Nugent et al., 2020). This was impeded in *Trem2* knock‐out mice, which resulted in an accumulation of cholesteryl ester and its oxidized form in the brain (Nugent et al., 2020). Understanding cholesterol regulation in microglia would open new avenues, particularly in terms of treatment for demyelinating diseases.

### 3.5 Sphingolipids

Sphingolipids are a very diverse class of abundant membrane lipids in the CNS; they are formed by de novo synthesis of sphinganine from serine and a long chain fatty acyl‐CoA and are divided into three main classes depending on their polar head group: glycosphingolipids, sphingomyelins and ceramides (Alaamery et al., 2021). Microglia are rich in sphingolipids (Fitzner et al., 2020) (Figure 3), particularly in brain regions and timepoints where altered microglial states (i.e., PAM or DAM) are found, such as the developing white matter, and during disease or injury conditions (Amat et al., 1996; Cammer & Zhang, 1996; Dehghan et al., 2022; Ellison & de Vellis, 1995; Muscoli et al., 2010; Pan et al., 2017; Saito et al., 2012, 2015; Wang et al., 2021).

Rat primary microglial cells incubated with gangliosides had increased levels of nitric oxide and pro‐inflammatory cytokines (Jou et al., 2006; Min, Pyo, et al., 2004; Min, Yang, et al., 2004; Pyo et al., 1999). Gangliosides contributed to activating the Janus‐kinase/signal transducers and activators of transcription (JAK–STAT) pathway in microglia via the accumulation of phosphatases in lipid membrane rafts (Kim et al., 2006). Furthermore, gangliosides administration facilitated the linkage of TLR2 to its adaptor protein, myeloid differentiation primary response protein (MYD88) (Yoon et al., 2008). Sulfatide, a galactosylceramide, was shown to induce nuclear factor kappa B subunit 1 (NF‐κB) stimulation in mouse primary microglial cells (Jeon et al., 2008). Delivering glycosphingolipid‐enriched exosomes intracerebroventricularly into an AD pathology mouse model led to their uptake by microglia and reduced Aβ accumulation, potentially though these exosomes acting as a scavenger for Aβ (Yuyama et al., 2014). It seems that the ganglioside types may influence microglial pro‐inflammatory cytokine release, with GM1, GD3, GD1a, GD1b, and GT1b reducing, and GM3 and GQ1b promoting this release (Galleguillos et al., 2022). GM1 administration also ameliorated TREM2‐mediated microglial phagocytosis, through activating CD33, as well as ameliorated chronic sleep restriction‐induced synapse loss and memory impairment (Tan et al., 2023).

This also appears to be true for ceramides. Rat primary microglial cells incubated with C8‐ceramide released brain‐derived neurotrophic factor (BDNF), without increasing pro‐inflammatory cytokine production (Nakajima et al., 2002). C2‐ceramide in rat primary microglial cells promoted prostaglandin E2 synthesis (Akundi et al., 2005), as well as increased levels of IL‐1β in mouse primary microglial cells (Scheiblich et al., 2017). β‐glucosylceramide accumulation in microglia in Gaucher Disease led to phagocytosis of neurons in a mouse model of the disease, as well as in surgical resection samples from patients (Shimizu et al., 2023). However, other studies have shown that administration of C2‐ (as well as C6‐ and C8‐)ceramides inhibited pro‐inflammatory cytokine production in rat primary microglial cells and LPS‐exposed mice; furthermore, C2‐ceramide exerts these effects via penetrating into microglia and contributes to interfering with TLR4 and LPS interactions (Jung et al., 2013).

A current understanding in the field suggests that there is a delicate balance between types of sphingolipids, as their ratio is altered in a number of neurodegenerative disease conditions (Alaamery et al., 2021; Allende et al., 2021; de Wit et al., 2020; Sood et al., 2023; Xiao, 2023; Yuan et al., 2023). More research is needed to determine the effects of modulating sphingolipids in microglia, throughout development and in the context of CNS pathological alterations.

### 3.6 Plasmalogens

Plasmalogens are a particular group of glycerophospholipids that contain a vinyl‐ether at the sn‐1 position in the glycerol backbone (Gorgas et al., 2006). This particular bond confers plasmalogens hydrophobicity and acid/oxidation lability (Gorgas et al., 2006). At the sn‐2 position, plasmalogens are usually esterified with a long chain of n‐6 or n‐3 PUFA (Gorgas et al., 2006). At the sn‐3 position, they are esterified to a phosphate group linked to an alcohol, being the most abundant ethanolamine (PlsEtn) and choline (PlsCho), which together represent the polar head (Gorgas et al., 2006; Vance, 2010). Plasmalogens with ethanolamine are particularly abundant in human brain, heart, eosinophils, and neutrophils (Braverman & Moser, 2012). Also, in mice, this type of plasmalogen is abundant in the brain (Braverman & Moser, 2012). They constitute up to 20% of the phospholipids in cell membranes (Braverman & Moser, 2012). They are part of subcellular membranes including the nucleus, endoplasmic reticulum, Golgi apparatus, and mitochondria (Braverman & Moser, 2012). Of note, the highest amount of plasmalogens in mammals is present in the myelin sheath (Dorninger et al., 2022).

The levels of plasmalogens are tightly regulated by their biosynthesis de novo and their degradation (Bozelli et al., 2021). CNS inflammation has been consistently related to lower levels of plasmalogens, although the cause of this reduction is still unknown (Bozelli et al., 2021). Plasmalogens can be targeted by ROS because of their high oxidative liability and cytochrome c in oxidative conditions; they can be substrates of phospholipase C which catalyzes the removal of the polar head and substrates of phospholipase A2 which leads to the generation of lipid bioactive molecules, commonly PUFA, such as n‐6 arachidonic acid and n‐3 DHA (Gorgas et al., 2006; Jenkins et al., 2018; Yang et al., 1996). Particularly, different inflammatory stimuli in vitro (i.e., LPS and IFNγ) induced the phosphorylation of the cytosolic phospholipase A2 in mouse primary microglial cells and this was required to increase ROS/nitric oxide production through the lipoxygenase pathway, showing a direct link between pro‐inflammatory stimuli and plasmalogen metabolism in the microglial pro‐inflammatory response (Chuang et al., 2015). Also, reduction of the enzyme involved in the biosynthesis of plasmalogens (*Gnpat* knock‐down) led to the activation of NF‐kB pathway of mouse primary microglial cells and enhanced the expression of pro‐inflammatory cytokines such as IL‐1β induced by LPS. In vivo, the knock‐down of *Gnpat* in mice resulted in more rounded microglial morphology and increased the expression of *Il‐1b* and *Mcp‐1* (Hossain et al., 2017). Indeed, pro‐inflammatory stimuli, such as LPS in the MG6 microglial‐like cell line and primary mouse microglial cells led to a reduction of *Gnpat*, while mice injected intraperitoneally with LPS for 7 days showed reduced hippocampal *Gnpat* expression (and levels of PlsEtn) along with NF‐kB activation in IBA1+ microglia and GFAP+ astrocytes, but not NeuN+ neurons (Hossain et al., 2017). These results provide in vitro and in vivo evidence that the levels of plasmalogens are related to glial reactivity to pro‐inflammatory stimuli. In fact, incubation with plasmalogens delayed the endocytosis of TLR4 in mouse primary microglial cells and the BV‐2 cell line, which could explain the reduced pro‐inflammatory response to LPS (Ali et al., 2019). On the contrary *Gnpat* knock‐down in BV‐2 cultures accelerated endocytosis after LPS treatment (Ali et al., 2019).

Administration of plasmalogens containing 47.6% PlsEtn, 49.3% PlsCho, 2.4% sphingomyelin, and 0.5% other phospholipids, prevented the increase in IBA1+ cells in the hippocampus observed when injecting LPS intraperitoneally for 7 days to induce CNS inflammation in mice (Katafuchi et al., 2012). Recently, a study showed that the intragastric administration of plasmalogens enriched with EPA, DHA, and other n‐3 PUFA for 2 months to 16‐month‐old female mice improved cognitive performance and overall physical appearance (absence of gray hair and their hair was glossier and thicker) (Gu et al., 2022). Plasmalogen supplementation also increased synaptophysin levels and the number of synapses in the hippocampus as well as restored microglial age‐associated morphological changes (Gu et al., 2022). Plasmalogen levels were found to be reduced in patients with AD, Parkinson's disease, autism spectrum disorder, and Down syndrome (Dorninger et al., 2022). Preclinical evidence points to plasmalogens as potential regulators of glial inflammatory functions and diet supplementation showed efficacy in counteracting the effects of their diminution both in models of pathology and aging, potentially through their actions in microglia.

## 4 CHALLENGES REGARDING METHODOLOGY TO STUDY MICROGLIAL LIPID METABOLISM

Assessing lipid metabolism in microglia is still a challenge, especially when it comes to understanding homeostatic and pathological conditions in vivo. Our current understanding of microglial lipid metabolism is mainly provided by experiments in culture which allow to study intracellular cascades in controlled conditions and response to different stimuli. However, microglial primary cultures, cell lines, or derived from human cells are devoid of their microenvironment and usually exhibit a more reactive phenotype far different from the microglia in vivo (Pesti et al., 2024). Data generated in vitro provides useful information as a starting point to disentangle microglial lipid metabolism. Regarding in vivo studies, microglia's role in brain metabolism and how microglial metabolism impacts their functions have been overseen for several years and most lipid studies are not microglia‐specific, for examples, staining for lipids or lipidomic of a whole brain area. Nowadays, scRNA‐seq allows for the analysis of transcripts related to lipid metabolism particularly in sorted microglial cells. Despite this advance, as stated before, a transcriptomic signature does not necessarily provide functional information. Another important consideration is that lipid metabolism is a dynamic process and many imaging and molecular approaches like lipidomics are unable to provide information about kinetics (Kim et al., 2020).

## 5 CONCLUSION

Lipid signaling and metabolism in microglia are gaining attention since many genes that are upregulated during development and pathological states pertain to lipid processing proteins or transporters (Keren‐Shaul et al., 2017; Li et al., 2019; Paolicelli et al., 2022). Indeed, lipids proved to shape microglial functions particularly their immune response and phagocytic activity. It is particularly interesting that diet or supplementation of different lipid mediators can influence microglial functions and impact beneficially or detrimentally the CNS across a lifetime. This could be because of a modulation of microglial metabolism which adapts to the available nutrients or through changes in microbiota composition (Erny et al., 2015; Thion et al., 2018). In addition, new treatment targets to modulate lipid metabolism could provide new treatment avenues for incurable pathologies, including AD and demyelinating diseases.

## 6 FUTURE DIRECTIONS AND PERSPECTIVE

Microglial states are broadening our view of their roles across physiology and pathology. In particular, states associated to pathology share as a common trait the convergence of lipid metabolism. The next step in the field is to understand how lipids modulate microglial functions considering their dynamics in vivo. Having a clear picture of lipid metabolism and signaling in microglia would allow us to propose targets to shift microglia to states that could be beneficial for a specific condition. Microglia have been shown to respond quickly to changes in lipid intake which is promising for potential treatments (Drougard et al., 2023). Moreover, we still have a long way to go to understand the regulation of lipid metabolism during development and aging, as well as how sex influences these processes.

## AUTHOR CONTRIBUTIONS

**Marianela E. Traetta:** Conceptualization; visualization; writing – original draft; writing – review and editing. **Haley A. Vecchiarelli:** Conceptualization; writing – original draft; writing – review and editing. **Marie‐Ève Tremblay:** Conceptualization; funding acquisition; supervision; writing – review and editing.

## CONFLICT OF INTEREST STATEMENT

The authors declare that the research was conducted in the absence of any commercial or financial relationships that could be construed as a potential conflict of interest.

### PEER REVIEW

The peer review history for this article is available at https://www.webofscience.com/api/gateway/wos/peer‐review/10.1111/jnc.16259.

## Data Availability

Data sharing is not applicable to this article as no new data were created or analyzed in this study.
